# Prevalence of crowding, boarding and staffing levels in Swedish emergency departments - a National Cross Sectional Study

**DOI:** 10.1186/s12873-020-00342-x

**Published:** 2020-06-18

**Authors:** Jens Wretborn, Joakim Henricson, Ulf Ekelund, Daniel B. Wilhelms

**Affiliations:** 1 Department of Emergency Medicine, Local Health Care Services in Central Östergötland, Linköping, Sweden; 2 Department of Clinical Sciences Lund, Emergency Medicine, Faculty of Medicine, Lund University, Lund, Sweden; 3 Department of Biomedical and Clinical Sciences, Linköping University, S58185, Linköping, Sweden

**Keywords:** Emergency department, Crowding, Boarding

## Abstract

**Background:**

Emergency Department (ED) crowding occurs when demand for care exceeds the available resources. Crowding has been associated with decreased quality of care and increased mortality, but the prevalence on a national level is unknown in most countries.

**Method:**

We performed a national, cross-sectional study on staffing levels, staff workload, occupancy rate and patients waiting for an in-hospital bed (boarding) at five time points during 24 h in Swedish EDs.

**Results:**

Complete data were collected from 37 (51% of all) EDs in Sweden. High occupancy rate indicated crowding at 12 hospitals (37.5%) at 31 out of 170 (18.2%) time points. Mean workload (measured on a scale from 1, no workload to 6, very high workload) was moderate at 2.65 (±1.25). Boarding was more prevalent in academic EDs than rural EDs (median 3 vs 0). There were an average of 2.6, 4.6 and 3.2 patients per registered nurse, enrolled nurse and physician, respectively.

**Conclusion:**

ED crowding based on occupancy rate was prevalent on a national level in Sweden and comparable with international data. Staff workload, boarding and patient to staff ratios were generally lower than previously described.

## Background

The emergency department (ED) is the nexus for patient inflow at a modern hospital. The combination of high acuity patients and frequent peaks in demand often results in crowding and a high workload for the staff [1, 2].

Crowding has been linked to increased inpatient mortality and decreased quality of care [3–6]. Many investigations have been conducted at single EDs or in local health care systems, with large variations in the extent of crowding, and nationwide data are lacking [6–8]. Patients waiting in the ED for an in-hospital bed, also known as boarding or access block, have been identified as an important factor for ED crowding [2] but prospective studies of the problems are scarce [9, 10].

Sweden, with a population of 10 million, has a universal publicly funded health care system granting emergency care with a small co-payment at 72 EDs spanning from small rural EDs to large urban academic EDs. Despite a long tradition of high-quality healthcare databases in Sweden, the emergency medicine register still lacks national coverage and includes limited data on crowding [11].

Crowding in Swedish EDs has previously been a limited problem [12], but news reports have raised the issue in recent years, and several research projects on the topic have been initiated [13]. Sweden lacks unified national information about ED attendances but based on government reports from 2010 and 2015, it is clear that ED attendances and waiting times have increased [14] Similar trends are seen in Denmark which has a comparable health care system [15]. With no proportional increase in hospital beds during the last 30 years, Sweden now has the fewest inpatient beds per capita of all OECD countries [16]. Based on the conceptual input-throughput-output model [17], there is a clear risk of crowding given the increasing number of ED attendances and decreasing number of hospital beds, limiting capacity to admit patients.

Despite almost two decades of international research, there is no consensus on how to measure crowding, and several methods have proven reliable and valid [5, 18]. In this study we chose to measure occupancy rate and staff workload to encompass different aspects of crowding [17]. Occupancy rate is a simple numeric variable that accounts for the core resource, an ED treatment bed. Staff perception of crowding or workload has been used to derive the International Crowding Metric in Emergency Departments (ICMED), National Emergency Department Overcrowding Score (NEDOCS) and Swedish Emergency Department Assessment of Patient Load (SEAL), but is less studied outside these scores [19–21]. Workload is subjective in nature, but has face validity as a measure of human resource utilisation and may complement occupancy rate at times when available treatment beds does not reflect crowding. An example could be a surge in high acuity patients at a period with low staffing, which will result in a high workload at a low occupancy rate.

We aimed to study the current levels of crowding at Swedish EDs by assessing patient attendance, occupancy rate, boarding as well as staff numbers and workload.

## Methods

### Study design and population

We conducted a cross sectional, multi-centre study during 24 h on April 25th 2018. All Swedish EDs listed in the national healthcare institution registry were offered to participate by written invitation (e-mail) to the officially listed head of department. The written invitation was followed up by a telephone call. Participation was confirmed in writing by the department head. EDs were classified by their hospital status in Sweden (Academic, Community and Rural), where academic centres were the only centres with tertiary, highly specialised care (such as neurosurgery, cardiothoracic surgery, transplantations and advanced burn care).

### Data collection

During the 24 h period, each ED collected data at five pre-specified time points (00:00, 06:00, 12:00, 18:00, 23:59). A questionnaire was supplied by the research coordination centre and the method of data gathering was left to each ED. We did not collect information on the personnel gathering the data. Data included the number of registered ED patients, the number of patients waiting for an in-hospital bed (boarding), the number of enrolled nurses, registered nurses and physicians, occupancy rate and overall ED workload. Each ED also provided information on the annual and daily census in the previous year (2017) and the number of available treatment beds.

### Measurements and definitions

We defined occupancy rate like McCarthy et al. [18] as the number of patients divided by the number of beds where basic care could be provided, excluding corridor spaces. An occupancy rate above 1.0 was set as the cut-off to indicate crowding. Workload was assessed on a graded Likert scale with anchors from 1 (very low workload) to 6 (very high workload). It was used as a measure of staff perception of crowding in the ED and a score of 4.5 or higher was considered to indicate crowding [20]. A boarding patient was defined as a patient with a decision for admission who was still present in the ED, regardless of the duration.

The EDs reported if the study period was representative in terms of workload and if there were any extraordinary events during the 24 h period. They also graded the supply of inpatient beds during the study period on a scale from 1 (good bed availability) to 10 (extreme bed shortage). Data was recorded prospectively on a paper-based report form by a senior staff member and subsequently submitted in a digital form to the study coordinator.

### Statistics

Census was reported as median. Registered patients, staffing levels and workload were reported as means with standard deviations (SD). Boarding patients were reported as medians with interquartile range (IQR).

Correlations were assessed using ordinary least-squares linear regression. To compare medians, the grand median for each group was calculated. A two by two table was created by classifying each value as above or below the grand median, and we then applied Fisher’s exact test. Staffing ratios were compared using parametric ANOVA and post-hoc testing with t-test. Boarding was compared using Kruskal-Wallis test and post-hoc Mann-Whitney-U test. The Holm method was used to adjust for multiple comparisons [22].

Data was imported into Pandas dataframes (version 0.23.4, https://pandas.pydata.org/) [23] and analysed with computer scripts in the Python programming language (version 3.7.2, https://www.python.org) using the scipy scientific library (version 1.1, https://www.scipy.org/) [24, 25] and statsmodels (version 0.10, https://www.statsmodels.org) [26] for statistical calculations.

### Ethics

This study was carried out in accordance with The Declaration of Helsinki [27]. This study was approved for all sites by the regional ethics review board in Linköping, Sweden (permit reference: 2018/50–31). Informed consent was waived since no identifiable personal data was collected.

## Results

### Participating sites

**Fig. 1:**
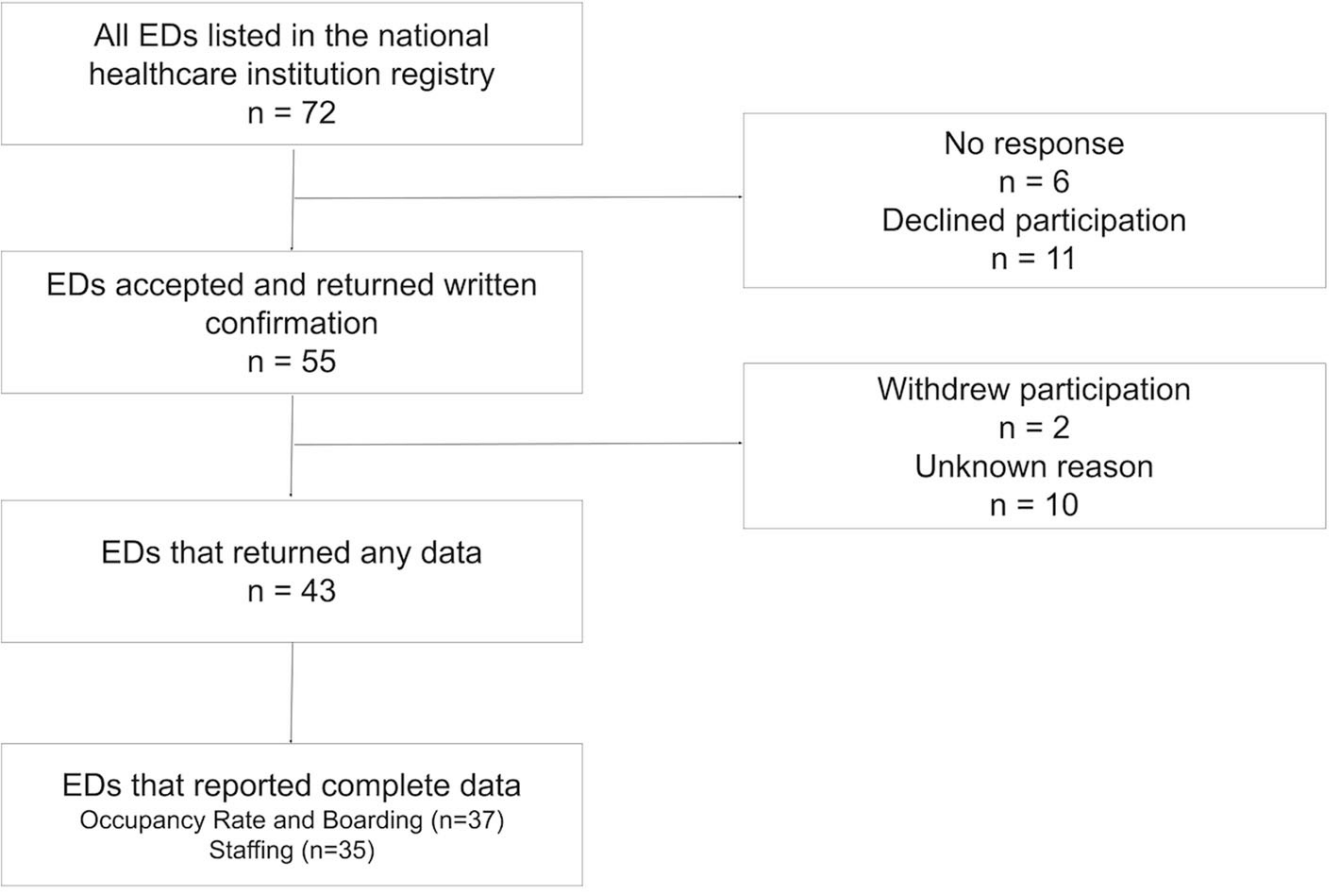
Flowchart of participating sites

**Fig. 2:**
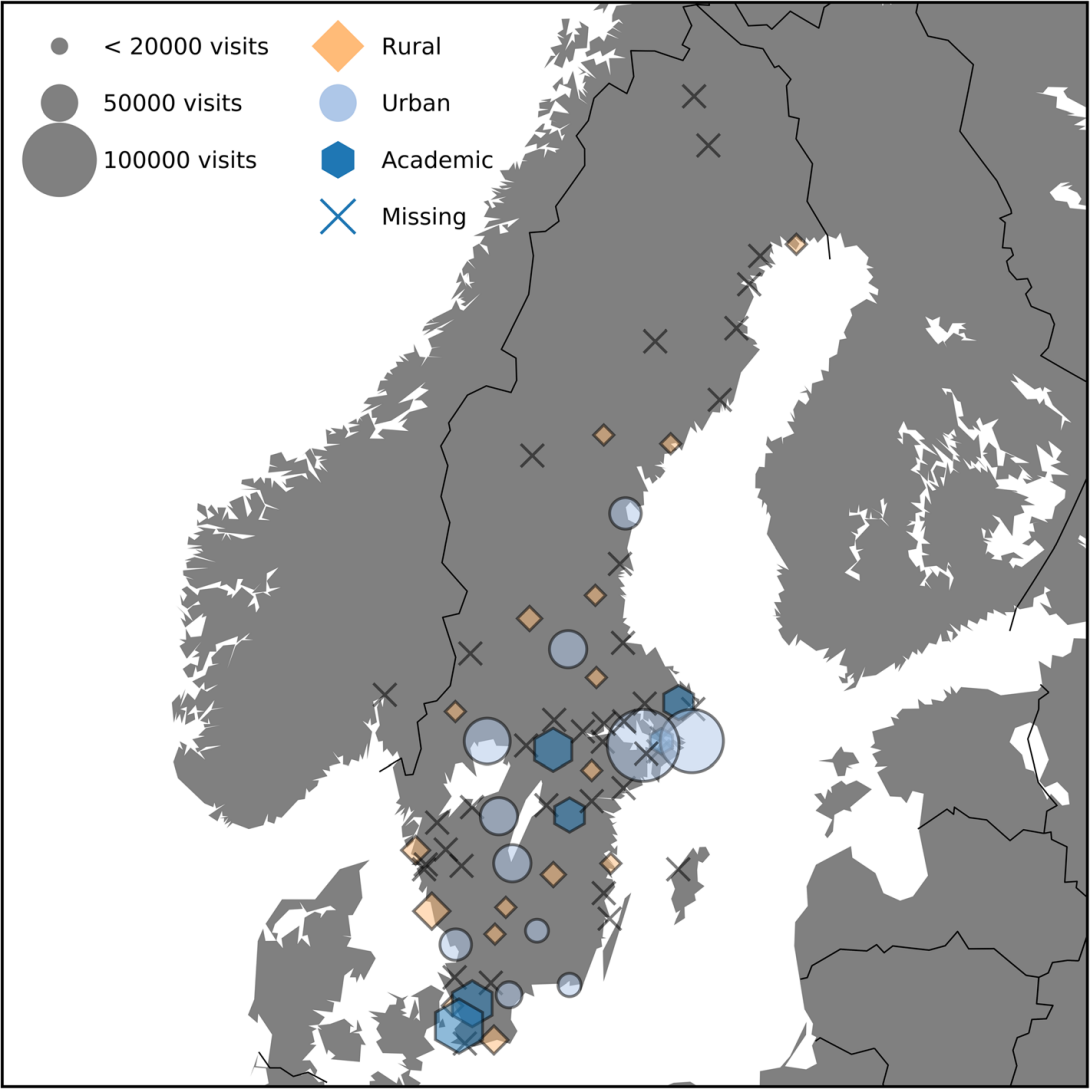
Map of Sweden and geographic distribution of enrolled and missing EDs

Fifty-five out of 72 eligible EDs accepted participation and 37 (51%) delivered complete data for the number of patients and workload assessments **(**Fig. 1**)**. Thirty-five (49%) EDs reported complete staffing data for all time points. Five out of Sweden’s 7 (71%) university hospitals responded in the study. The geographic distribution of the responding EDs is shown in Fig. 2.

The median number of annual visits in the participating EDs were 35,000 (range 3300–102,000) with 15 (44%) reporting more than 40,000 visits per year. The number of patients seen in the EDs during the 24 h period was not different compared to the daily census of the previous year (median 95 vs 93, *p* = 1.00).

### Registered patients and boarding

**Fig. 3:**
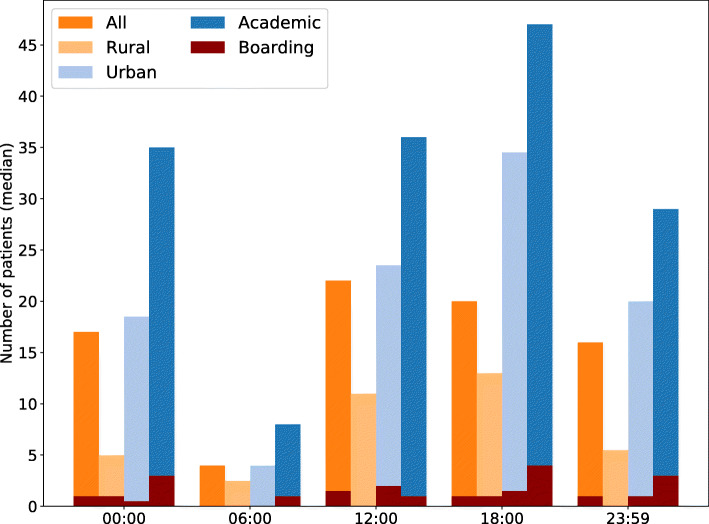
Number of patients present and boarding for each hospital type at each time point

The number of registered patients showed a diurnal pattern in most EDs with a median of 20 (IQR 14–41) patients present at 18:00 and 4 (IQR 2–6) patients at 06:00. The number of patients boarding in the ED followed the same pattern (Fig. 3) and correlated modestly to the number of patients in the ED (r^2^ = 0.31). Boarding was more prevalent in academic EDs than rural EDs with a median boarding of 3 (IQR 1–4) and 0 (IQR 0–1) patients respectively (*p* = 0.008). There was no significant difference between urban EDs (median 1, IQ 0–2) and rural or academic EDs.

### Occupancy rate and workload

**Fig. 4:**
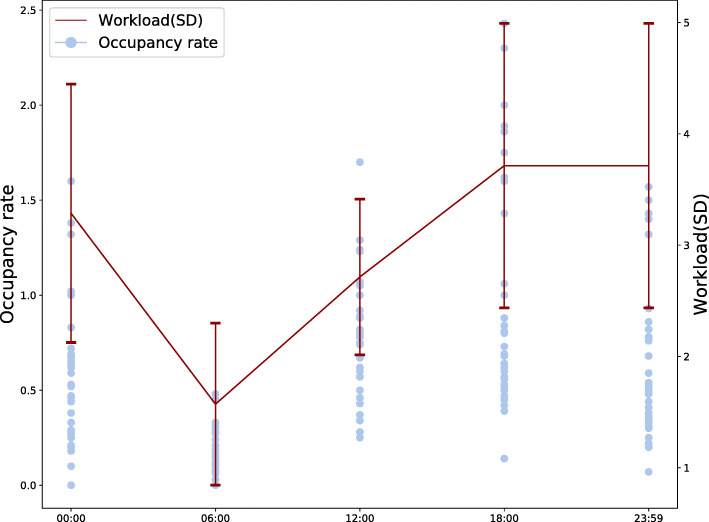
Occupancy rate in relation to workload at each time point

Occupancy rate was greater than 1.0 on at least one occasion at 12 EDs (37.5%) and on a total of 31 out of 170 (18.2%) time points. Mean occupancy rate was higher in academic EDs compared to rural EDs (0.89 vs 0.45, difference 0.37, 95%CI 0.16–0.58, *p* < 0.001) and for urban compared to rural EDs (0.54 vs 0.45, difference 0.24, 95%CI 0.016–0.48, *p* = 0.037) but there was no significant difference between academic and urban centres (*p* = 0.45). Mean workload was 2.65 (±1.25) and as higher than 4.5 at 14 out of 170 time points (8.2%). There was a moderate correlation between workload with occupancy rate (*r*^*2*^ = 0.36) and assessed workload showed a similar diurnal pattern as occupancy rate (Fig. 4).

### Staffing levels

**Fig. 5:**
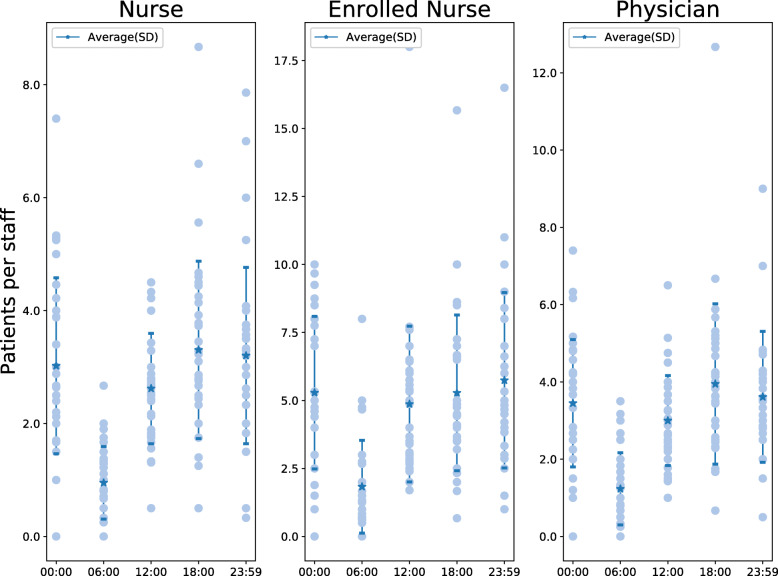
Patient to staff ratio for each staff category at each time point

During the 24 h period, there was an average of 2.6 (±1.6) patients in the ED per registered nurse, 4.6 (±3.1) per enrolled nurse and 3.2 (±2.2) per physician, with little difference between time points except 06:00 which had lower ratios for all providers (Fig. 5). There were more patients per nurse in academic compared to rural EDs (4.4 vs 2.2, *p* = 0.02) but not compared to urban EDs (4.4 vs 3.2, *p* = 0.08) and no difference between rural and urban EDs (2.2 vs 3.2, *p* = 0.13). There were more patients per physician at academic than rural EDs (4.4 vs 2.6, *p* = 0.01), but there was no difference compared to urban EDs (4.4 vs 3.3, *p* = 0.13) or between urban and rural EDs (3.3 vs 2.6, *p* = 0.13).

### Non-clinical events

There were no extraordinary incidents reported in any of the participating EDs’ catchment areas. Four sites (11%) reported hospital-specific disturbances. Of these, two were related to downtime in the electronic health records (EHRs) and two due to disturbances in other digital support systems (ancillary testing, registration and internal telephone system). None of these events were reported to affect the ED workflow.

## Discussion

In this national cross-sectional study at Swedish EDs during 24 h, we provide a snapshot of current Swedish ED crowding, boarding and staffing, which has never been done before. We observed that boarding was common and occupancy rates were generally high, primarily in academic EDs but also in urban EDs. On average, patient to staff ratios for nurses were on par with internationally reported levels (see below), but lower for physicians. There were more patients per staff at academic centres compared to rural hospitals. Workload was mostly perceived as low to moderate, which indicated limited staff problems related to crowding during the study period.

Occupancy rate correlated modestly with workload, which suggests that these may reflect different aspects of crowding. Workload was subjectively assessed by a single senior provider at each ED which limits the generalizability. However, subjective provider judgement was used as an outcome measure in the original NEDOCS trial and this has been validated in several different settings [19, 28, 29]. Physicians’ judgment has also proved to be equal or superior to structured decision support tools in many types of clinical decision-making ranging from imaging in trauma to the investigation in suspected pulmonary embolism [30].

Boarding was prevalent at many sites during this study. Generally however, boarding was reported as lower compared to the limited data from the United States (US) and Australia published so far. In a US cross sectional study of 89 EDs, 22% reported boarding patients and 73% of EDs had more than 2 patients boarding [9]. In a registry study of 139,509 ED visits in the US, median boarding time was 79 min [31]. In a study of 72 EDs in Australia, boarding ranged from 2 to 22 patients at two time points [10]. The difference in findings between the present and previous studies may be due to sampling errors or temporal effects, but it may also reflect possible differences in health care systems. Lack of inpatient beds is usually the basis for boarding patients in the ED. Since Sweden has fewer inpatient beds per capita than the US and Australia, our results support the claim that boarding may not be directly related to the number of hospital beds, but also to resource utilisation [32], both in single hospitals and in the system as a whole. It is important to note that our definition of boarding did not include a minimum waiting time after the decision to admit, and that we did not gather any further information regarding the admissions.

The staffing ratios were comparable at all study sites with most variation observed around midnight. This finding likely reflects that staffing is reduced at night-time and that staffing ratios therefore become more dependent on the inflow of patients. We did not collect information about work shifts at each ED and cannot exclude that this may explain some of the variation in staff ratios. The emergency medicine literature provides little data for comparison, but Schneider et al. reported higher mean ratios for nurses (4.2) and physicians (9.7) in the US in 2003 [9]. The difference, particularly for physicians, may partly be due to different denominators since we registered all physicians irrespective of training level in this study. In Sweden, a majority of the current ED physicians are pre-interns, interns or residents and only a minority are on site consultants [14]. This may result in higher numbers of physicians working in the ED compared to the US, where EDs are primarily staffed by residents and consultants. There are no national recommendations for staffing ratios in Sweden but our results are within the *four patients to one nurse* ratio legislated in the US state of California [33].

During the 24 h period, four study sites (11%) noted disturbances in the EHR or support systems, and this has previously been associated with increased ED crowding [34]. All EDs in Sweden use EHRs with a range of digital support systems for radiology, laboratory and other ancillary facilities. The reports may thus be an indicator of the fragility of complex digital systems to which ED providers must adapt. The lack of adverse events suggests mature systems with some resilience against unexpected downtime, leading to no serious disruption of clinical work. However, further studies will be needed to determine the frequency of EHR disturbances and their effects on emergency care.

### Limitations

This was a cross sectional study during only 24 h, and the generalisability of the results is therefore limited. There may be both seasonal differences in the demand and availability of healthcare resources. However, given the range of EDs both in size and geographic location, we believe that the results are a representative snapshot of the ED situation on a national level in Sweden.

The response rate was 51% among the eligible EDs regarding patient and crowding data, which is quite high compared to similar studies. Again, generalisability was most likely increased by the wide range of ED size and location. However, the fact that so many EDs chose to not participate emphasizes the need for mandatory and public reporting of this type of information for all EDs.

## Conclusion

Based on this cross sectional study during 24 h in 37 EDs, crowding as measured by occupancy rate and ED boarding is prevalent in Sweden. Occupancy rates were comparable with international data, whereas boarding and patient to staff ratios were lower than reported in the limited existing literature. In contrast to occupancy rate and boarding, patient to staff ratios and perceived workload did not suggest high levels of crowding. These observations highlight the importance of measuring different aspects of the complex entity of ED crowding.

## Data Availability

The datasets generated during and/or analysed during the current study are available from the corresponding author on reasonable request.

## Abbreviations

ED: Emergency Department
EHR: Electronic Health Record
ICMED: International Crowding Metric in Emergency Department
IQR: Interquartile Range
NEDOCS: National Emergency Department Overcrowding Score
SEAL: Swedish Emergency Department Assessment of Patient Load
SD: Standard Deviation
US: United States
